# Medical Psychotropics in Forensic Autopsies in European Countries: Results from a Three-Year Retrospective Study in Spain

**DOI:** 10.3390/toxics10020064

**Published:** 2022-02-01

**Authors:** Maira Almeida-González, Luis D. Boada, Luis Alberto Henríquez-Hernández, Octavio P. Luzardo, Enrique Zaragoza, Guillermo Burillo-Putze, María P. Quintana-Montesdeoca, Manuel Zumbado

**Affiliations:** 1 Institute of Legal Medicine of Las Palmas, Paseo Blas Cabrera s/n, 35016 Las Palmas de Gran Canaria, Spain; maira.almeida@ulpgc.es (M.A.-G.); luis.boada@ulpgc.es (L.D.B.); eazpharry@yahoo.es (E.Z.); 2 Toxicology Unit, Research Institute of Biomedical and Health Sciences (IUIBS), Universidad de Las Palmas de Gran Canaria, Paseo Blas Cabrera s/n, 35016 Las Palmas de Gran Canaria, Spain; octavio.perez@ulpgc.es (O.P.L.); mariadelpino.quintana@ulpgc.es (M.P.Q.-M.); manuel.zumbado@ulpgc.es (M.Z.); 3 Emergency Department, Hospital Universitario de Canarias, Carretera Ofra S/N, 38320 San Cristóbal de La Laguna, Spain; gburillo@ull.edu.es

**Keywords:** legal psychotropics, benzodiazepines, opiates, antidepressants, forensic autopsies, medico-legal issues

## Abstract

Medical psychotropics are widely used and prescribed in developed countries. These medications may have an impairing effect on mood or perception and may induce harmful behaviors. Nevertheless, in Europe, studies on their importance from a medico-legal perspective are scarce. To fill this gap, we evaluate the determinants of these drugs in a retrospective study based on data obtained from forensic autopsies. Toxicological analyses were performed on 394 blood samples from compulsory autopsies at the Institute of Legal Medicine of Las Palmas. Of the samples, 41% (159) were positive for at least one psychotropic, with benzodiazepines being the most frequently detected (24.1%), followed by opiates and antidepressants. Benzodiazepines, opiates, and antidepressants were detected more frequently in men who suffered a violent death. More than 30% of the positive samples showed two or more drugs, suggesting a prevalence of polypharmacy among forensic autopsy subjects, with the most frequently combination found being benzodiazepines plus opiates (28.3% of positive samples). A combination of opiates plus antidepressants was also found in subjects involved in violent deaths. Our results suggest that more than 40% of the adult European population involved in medico-legal issues may be under the influence of legal psychotropics. The link between violent deaths and the use of medical psychotropics is particularly worrisome and indicates that these drugs should be carefully monitored in developed countries, in all forensic autopsies, in a similar way to illegal psychotropics.

## 1. Introduction

Medical psychotropics are drugs that act on the central nervous system (CNS), producing several effects, such as excitation, hallucinations, and/or depressant/sedative effects. The group of psychotropic medications includes benzodiazepines (BZDs), barbiturates, Z-drugs, antidepressants (ADPs), antipsychotics, and medical opiates (MOs). They can induce temporary changes in attention, perception, mood, consciousness, and behavior, putting users at a higher risk of being involved in medico-legal issues (cases in which the toxicological findings will have not only medical, but also legal repercussions). Therefore, the legal consequences related to their use could be of great relevance because their use (or misuse) could be related to traffic or occupational accidents, suicides, homicides and so forth [1,2]. In fact, medical psychotropics are frequently found in routine toxicological analyses at compulsory autopsies [3]. The problematic use of psychotropic drugs has increased dramatically in developed countries and, due to their effects on the CNS, an increased presence and involvement of these drugs in forensic issues seems evident [4,5].

In this regard, in 2014, 32% of suicides tested positive for opiates in the United States [6]. Furthermore, in Australia, BZDs were described as being found in 13.2% of homicide and suicide deaths [7]. Moreover, opiates have been reported to be positively associated with homicide and suicide by firearm, and antidepressants with hanging [8]. More recently, an association between medical prescription of BZDs and an increased risk of suicide has been described [9,10]. Interestingly, it has been reported that patients who died by suicide were prescribed more psychotropic medications than the general population, including antidepressants, anxiolytics, and hypnotics [11]. This increase in the use of psychotropics, especially BZDs and ADPs, is particularly pronounced in higher-income countries [12]. This phenomenon could be explained by an increase in the prevalence of common mental disorders, an increase in the intensity and duration of treatment and a higher number of therapeutic indications, or even by the growing access to these drugs without a prescription [13]. Thus, the use of ADPs has increased in European countries such as Germany, with a growth of 46% between 2007 and 2011 [12].

Regarding Spain, the Organization for Economic Co-operation and Development (OECD) statistics for 2015–2017 show that the volume of sales of anxiolytics and ADPs in outpatient services was high compared to the rest of the 25 OECD countries [14]. In this context, far from decreasing, the overall consumption of sedatives and hypnotics and even ADPs is currently increasing in Europe [15,16]. In fact, many studies seem to indicate that prescription drug abuse has reached an epidemic level, with opiates being the most abused type of prescription drug [17]. After opiates, the most abused prescription drugs are tranquilizers [18]. Like any other substance abuse disorders, prescription drug abuse has been associated with psychiatric illness, violence, and stress, which increases its importance in medico-legal issues [19].

In addition to this situation, the phenomenon of polypharmacy among psychotropic users makes the study of the determinants and importance of these drugs from a medico-legal perspective more complex. Polypharmacy, defined as the combination of several psychotropics drugs [20], is a very worrying phenomenon because it could be associated with an additional burden of adverse effects [21,22]. In this regard, the increasing rate of combined use of MOs and BZDs is of particular concern and is considered a major public health problem in the United States and in most developed countries [23,24].

Consequently, the relevance of these medical drugs from a forensic medicine perspective cannot be ignored. On the contrary, considering this situation, it should be mandatory in developed countries to know the impact of the use of medical psychotropics, alone or in combination, on medico-legal issues. This review of the published literature, and the results from our work based on data obtained from forensic autopsies in Spain, aims to gain a better understanding of the prevalence and forensic importance of the legal use of psychotropic drugs in European countries.

## 2. Materials and Methods

### 2.1. Study Population

This study was conducted in the Canary Islands, one of the so-called Outermost Regions of the European Union. The Canary Islands are in the Atlantic Ocean, 1600 km away from the southwest of Spain and only 100 km from the nearest point of the North African coast (southwest of Morocco). The archipelago has a resident population of about 2 million inhabitants and a visitant population of about 12 million people each year (mostly European people from United Kingdom, Germany, and Sweden). The Institute of Legal Medicine of Las Palmas serves a population of 1.1 million inhabitants of the province of Las Palmas [25,26].

A total of 402 forensic autopsies on subjects from European Union were performed at the Institute of Legal Medicine of Las Palmas in the study period (between January 2015 and December 2017). In one case, there was insufficient blood sample volume for toxicological analysis, in five cases the deceased may have been treated with psychotropic drugs (prior to death), and two cases involved subjects under 18 years of age. Consequently, the results of 394 forensic autopsies on adult subjects from European Union were finally included in the present work. Notably, 298 individuals were males (75.6%), and 96 females (24.4%). Only 39.6% of the subjects were younger than 45 years (official data in Spain show that the use of psychotropic drugs increase dramatically over 45 years old) [27], and 152 individuals were victims of violent death, including in this subgroup deaths by firearms, bladed weapons, drowning, fights and unintentional falls [28]. Table 1 summarizes the demographic and forensic characteristics of the study population.

### 2.2. Toxicological Analysis

Quantitative determination of psychoactive drugs in the blood samples was performed. At least 1 mL of blood was taken from the femoral vein at the time of autopsy. When this was not possible, the blood was taken directly from the heart. The availability of the sample depended on the decision of the coroner, depending on the circumstances of the autopsy. In general, the bodies were in a good state of preservation and kept refrigerated until the time of the autopsy. A minimum of 1000 μL was required for chromatographic analysis. Blood samples were kept at −7 °C until analysis. All samples were analyzed within 4 weeks of recovery. Socio-demographic characteristics of the subjects—age, gender, date, and cause of death—were obtained from forensic reports and studied in relation to the toxicological analyses. The analysis of the investigated drugs was performed by liquid chromatography coupled to mass spectrometry (LC/MS).

Because in forensic toxicology the biological sample is usually scarce, we selected several psychotropics (and metabolites) frequently consumed in Europe that can be measured simultaneously in the same chromatographic method. The drugs analyzed were: (a) BZDs, such as short-acting (half-life < 8 h): midazolam; intermediate-acting (half-life 8–24 h): alprazolam, bromazepam, clonazepam, lorazepam, oxazepam, temazepam, lormetazepam; long-acting (half-life > 24 h): nordiazepam, clordiazepoxide, diazepam, and flurazepam; (b) Z drugs: zolpidem and zoplicone; (c) ADPs, tricyclic- and tetracyclic-ADPs: amitriptyline, nortriptyline, and maprotiline; selective serotonin reuptake inhibitors (SSRI)-ADPs: paroxetine, sertraline, and citalopram; (d) MOs: morphine, tramadol, codeine, fentanyl, and methadone; (e) antipsychotics: haloperidol, olanzapine, quetiapine, and risperidone; and (f) barbiturates: pentobarbital, phenobarbital, thiopental, secobarbital, and amobarbital.

Drug-specific standards were supplied by Sigma (Sigma-Aldrich, Alcobendas, Spain). Solvents (acetonitrile and methanol), formic acid, and ammonium formate were of LCMS quality and were purchased from Merck (Darmstadt, Germany). Ultrapure water (UP) was produced in the laboratory using a Milli-Q Gradient A10 apparatus (Millipore, Molsheim, France). PFTE 0.22 μm syringe filters were purchased from Macherey-Nagel (Düren, Germany). Quality controls were supplied by LGC Standards, Barcelona, Spain.

After a solid-phase extraction procedure, the blood sample extracts were analyzed to identify and quantify the investigated substances using a UHPLC model 1290 (Agilent Technologies, Palo Alto, CA, USA), interfaced to an Agilent 6460 Triple quadrupole mass spectrometer, equipped with a jet stream electrospray interface operating in positive ionization mode (Agilent Technologies). The MS/MS analyzer parameters were: gas temperature 180 °C; gas flow rate 8 L/min; nebulizer 35 psi; sheath gas temperature 400 °C; sheath gas flow 12 L/min; capillary 3500 V. Data acquisition and peak areas were quantified using the Agilent MassHunter Workstation software package (MassHunter Quantitative Analysis Version B.07.01). A 5-point standard curve from 1.56 to 400 ng/ml of each analyte plus one negative and two positive controls were included in each analysis. Chromatographic separation was performed using an Agilent Poroshell 120 EC-C18 (2.1 × 100 mm, 2.7 µm), with a guard pre-column (2.1 × 5 mm, 1.8 µm) and a pre-filter. The column oven was set to 50 °C. The mobile phases were 2 mM ammonium formate and 0.1% formic acid in ultrapure water (A) and 2 mM ammonium formiate 0.1% formic acid in MEOH (B). The binary gradient was set as follows: 95% A min 0, 80% A min 1; 15% A min 12; 15% A min 14; 0% A min 14.01; 0% A min 16; 95% A min 17. The flow rate was 0.4 mL min^−1^. The volume injected was 2 µL and the total run time was 21 min. Frequency of detection, concentrations (mean and standard deviation, and median and 25th and 75th percentile), limits of detection (LODs) and quantification (LOQs) of the above cited analytes are shown in Table 2.

### 2.3. Statistical Analyses

Standard descriptive statistics (mean values and standard deviations, medians, 25th and 75th percentiles of the distribution, and frequencies) were calculated for the drugs measured. Categorical variables were summarized using absolute frequencies and percentages. The equality of proportions of the categories was contrasted with the non-parametric binomial and Chi-square tests. A *p*-value < 0.05 (two-tailed) was considered to be statistically significant. Database management and statistical analyses were performed using IBM SPSS Statistics v 27.0 software (IBM Co., Armonk, NY, USA).

## 3. Results

This study included results on the presence/absence of medical psychotropics drugs (excluding ethanol) in blood samples from the 394 compulsory forensic autopsies on adult subjects performed during the years 2015 to 2017 at the Institute of Legal Medicine of Las Palmas (Canary Islands, Spain). Our results and in-depth review of the literature on this topic provide important new insights on the prevalence and determinants of psychoactive drugs, taken alone or in combination, in a European adult population involved in medico-legal issues.

As shown in Table 1, the study population was predominantly older than 45 years (60.4%), and the mean age was 49.3 ± 13.1 years old. Among the deceased, 298 (75%) were males, and only 96 (24.4%) were females. The subjects were victims of traffic accidents in 10.7% of the cases, while 38.6% were classified as victims of violent death (including firearms, drowning, falls and overdose). The group of other decedents, which includes victims of heart diseases, infections, gastrointestinal diseases, sudden death, and unknown causes, includes 122 subjects (31%). The decedents were evenly distributed throughout the years included in the study, 2015, 2016, and 2017.

As shown in Table 3, 159 decedents (40.4% of the total samples included in the study) showed detectable levels of at least one psychotropic (≥1), with BZDs being the most frequently detected drugs (24.1% of the samples): intermediate- and long-acting-BZDs showing a higher frequency of detection than short-acting-BZDs, although it should be noted that only one short-acting-BZD was included in our toxicological analyses (midazolam).

In the study, as shown in Table 3, BZDs were detected more frequently in men (66.3% of the subjects) than in women (33.7% of subjects), especially in the case of intermediate-acting BZDs (*p* = 0.029), whereas long-acting BZDs were detected similarly in both genders. Nevertheless, it has drawn our attention that the use of short-acting BZDs seems to be more frequent in older people (≥45 years; *p* = 0.013), but not in the case of intermediate- and long-acting BZDs which seem to be used similarly by both age groups. There is a positive association between BZD use and violent death. It is worthwhile noting that MOs were also frequently identified (22.8% of the samples). As with BZDs, MOs were detected more frequently in men compared to women (*p* = 0.004), and in those over 45 years of age compared to younger people (*p* = 0.045). As in the case of BZDs, there is a clear positive association between MOs use and violent death (*p* < 0.001).

ADPs were also detected more frequently in men (56.1%) than in women (43.9%), and in those over 45 years of age than in younger people (*p* = 0.028). This pattern of consumption was evident for both types of ADPs analyzed: the classical ADPs (tricyclic and tetracyclic antidepressants), and the new ADPs (SSRI-ADPs). As in the case of BZDs and MOs, there is a clear positive association between ADPs use and violent death (see Table 3).

In contrast, barbiturates and Z-drugs were detected in a low number of samples (0.25 and 2%, respectively). As expected, antipsychotics were only detected in 16 decedents (4.1% of the samples), presumably psychiatric patients under medical treatment.

**Figure 1:**
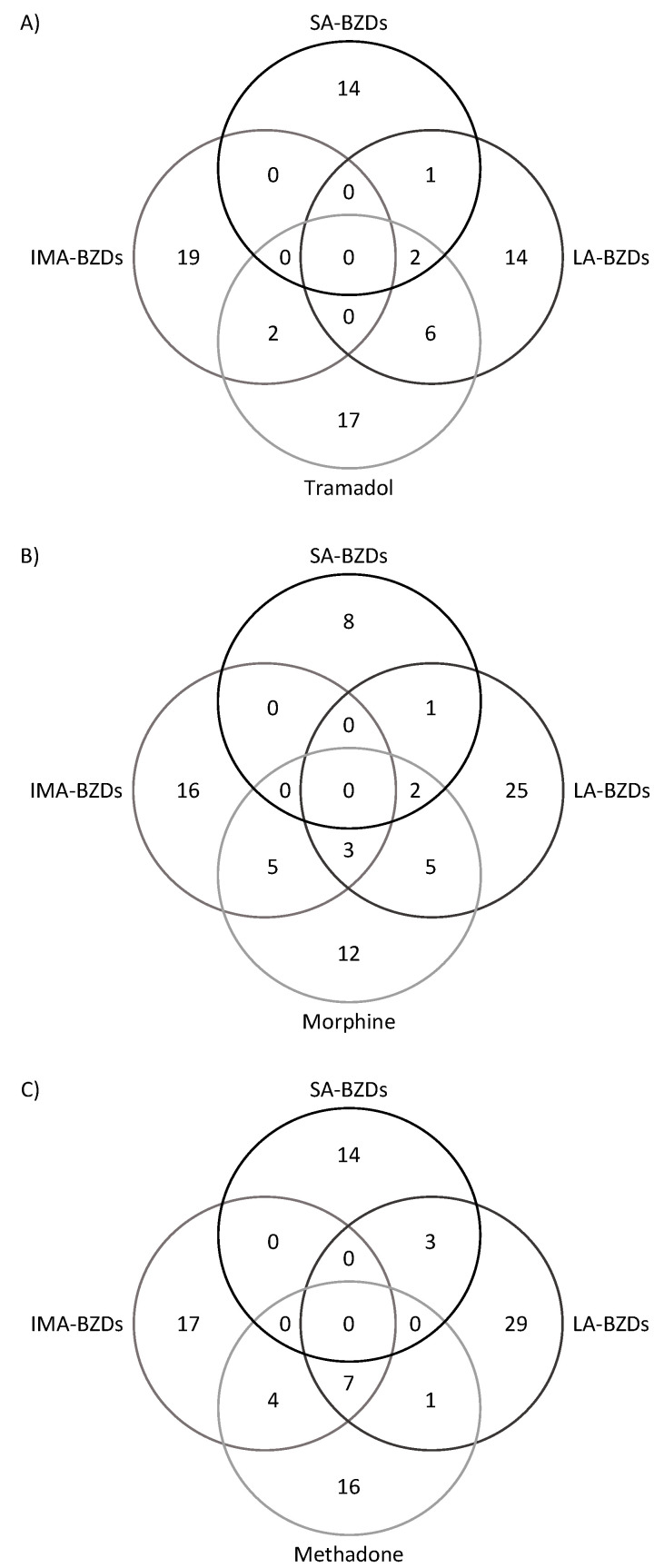
Shows the numbers of deceased testing positive for the most frequently found combinations of opiates. The number in the intersections shows the number of deceased positive for more than one class of substance. (**A**): Shows the numbers of deceased positive for tramadol and short-acting benzodiazepines (SA-BZDs), long-acting benzodiazepines (LA-BZDs), and intermediate-acting benzodiazepines (IMA-BZDs). (**B**): Shows the numbers of deceased positive for morphine and LA-BZDs, IMA-BZDs, and SA-BZDs. (**C**): Shows the numbers of deceased positive for methadone and LA-BZDs, IMA-BZDs, and SA-BZDs.

However, it is striking that 92 individuals had residues of more than one psychotropic in the blood (23.3% of the total samples), and that 31.4% of the positive samples had more than two psychotropic residues in the blood (Table 3). These results suggest that a significant percentage of psychotropic users consume several drugs simultaneously (polypharmacy). As shown in Table 3, polypharmacy (subjects showing ≥ 3 detected drugs) appears to be associated with violent death (*p* = 0.001). This is the case for MOs plus ADPs (mainly due to consumption of SSRIs-ADPs; *p* = 0.035). Interestingly, as shown in Table 4 among positive samples (*n* = 159) the most frequent combination found was BZDs plus MOs (45 samples; 28.3% of the positive samples), followed by BZDs plus ADPs (24 samples; 15.1% of the positive samples). It is worth noting the high frequency of detection of tramadol plus BZDs, with higher rates than methadone (Figure 1). As shown in Figure 1, in these combinations, long- and intermediate-acting BZDs were similarly involved. Thus, long-acting BZDs were detected in 30 decedents, and, among them, 19 were also concurrent users of intermediate-acting BZDs (Figure 1). In any case, as shown in Figure 1, it is worth mentioning at this point that the most frequently detected opiates (tramadol, morphine, and methadone) were mainly co-consumed with intermediate-acting- and long-acting-BZDs, while, on the contrary, the combination of these MOs with short-acting BZDs seems to be unusual.

## 4. Discussion

To date, a limited number of studies assessing the relevance of psychotropic medications in medico-legal issues have been published in Europe, except for studies analyzing drivers involved in road fatalities [29,30,31]. Nevertheless, it should be noted that most studies on drivers only assessed the prevalence of drugs belonging to the core list of substances included in the project “Driving Under the Influence of Drugs, Alcohol and Medicines in Europe” (DRUID Project) [32,33]. In any case, the DRUID Project does not monitor the prevalence of a group of widely prescribed benzodiazepines, antidepressants and opiates (i.e., BZDs such as lormetazepam; ADPs such as paroxetine; and MOs such as fentanyl), which have therefore been monitored in the present study. Although the results from our study do not indicate that the presence of these psychotropics is significant in road fatalities, it should be noted that an increased crash risk has been reported in drivers using short- and long-acting BZDs, antidepressants, high-potency opiates, and antipsychotics [34]. In any case, our results agree with those published by Herrera-Gómez et al. [30] and Domingo-Salvanya et al. [35] who did not find a high level of BZDs in Spanish drivers. The absence of association at this point could be explained by the fact that, in our study, road fatalities included not only drivers, but also passengers or even fatally injured pedestrians.

In other hand, it has been reported that the most frequently detected substances in forensic autopsies were benzodiazepines, opiates, and alcohol. Thus, such a situation has been reported in Norway [36], Japan [3] and even in some South American countries [37]. The results of the present study reinforce these reports, as in our series BZDs and MOs were also the most frequent medical psychotropics found in toxicological analyses of compulsory autopsy samples.

In addition to the utility for forensic purposes, it has been proposed that forensic-toxicological detection of pharmacological substances post mortem could be an indicator of ongoing pharmacotherapy in a region, as stated by Forsman et al. [38] and Hedlund et al. [39], who conducted pharmacoepidemiological studies in Sweden. Qualitative pharmacoepidemiological studies in relation to forensic toxicological findings may be especially useful in those drugs with low postmortem redistribution, because postmortem redistribution can cause an artificial increase/decrease in postmortem blood concentrations [40]. Thus, in drugs with low postmortem redistribution, the precision of forensic-toxicological findings may be high. This seems to be the case for the BZDs, MOs and antipsychotics measured in our study, which show a low postmortem redistribution as reported by Mantinieks et al. [40], Gleba et al. [41], and Nedahl et al. [42]. However, in addition to BZDs and MOs, such pharmacoepidemiological studies may also be useful to assess ADP consumption in a population as these drugs also seem to show adequate postmortem redistribution data as reported by Brockbals et al. [43] Consequently, our toxicological findings from forensic autopsies during the period 2015–2017 in the Spanish archipelago of the Canary Islands could be indicative of the consumption rates of these drugs in Spain. In this sense, our results, showing that BZDs were the most frequently detected psychotropics, followed by MOs and ADPs, suggest a high rate of consumption of these psychotropics, mainly BZDs. These results are consistent with previous studies indicating that BZDs are among the most commonly used drugs in the general population of developed countries, mainly due to the diverse range of indications, e.g., they are commonly prescribed in high-income countries for insomnia, anxiety disorders, acute alcohol withdrawal, muscle spasticity, and so forth [44,45]. Furthermore, the results of our study, which show that about 25% of samples from compulsory autopsies were positive for these drugs, agree with those population-based studies that reported a high prevalence of benzodiazepine use ranging from 2.1% up to 5.2% in the general population, and among older populations with a prevalence ranging from 30.0% to 35.1%. In addition, our results reinforce the data described in Spanish population showing a high rate of consumption of these psychoactive/psychotropic drugs in subjects older than 45 years [46,47]. Far from diminishing, nowadays the COVID-19 pandemic may be inducing increasing rates of psychotropic drug use, as the current outbreak of COVID-19 disease has profoundly changed the lives of many people worldwide, with studies suggesting an increasing burden of poor mental health, such as anxiety and depressive disorders, insomnia, etc. [48,49,50]. In this scenario, a greater relevance of psychotropics from a forensic perspective is expected.

Nevertheless, our results, linking BZD consumption to violent death, highlight the relevance of these drugs in medico-legal issues. In fact, BZDs have been a focus of concern in recent years, not only because of their widespread use but also because of their association with dependence and withdrawal reactions after discontinuation, as well as several possible consequences, such as an increased risk of traffic accidents, falls and cognitive impairment, especially among elderly users [51]. Thus, our results, which show a clear association between BZD use and violent deaths (including falls and overdose) in decedents older than 45 years point to the possibility that the role played by these drugs is highly relevant in deaths requiring compulsory autopsy. Furthermore, it has been reported in developed countries that the number of deaths involving BZDs has almost doubled in the last decade [44,51]. Although in our study there was no association between suicide and BZD use, such use has been reported to be a known risk factor for self-harm and suicide [52,53]. Perhaps the relatively low number of suicide victims in our series (19.8%) could explain this lack of association. In addition, the recreational use of BZDs, especially in combination with opiates, has received the attention of researchers and physicians since the 1970s. Drug users seem to have discovered that BZDs are able to enhance the positive subjective effects of opiates [24]. Today, abundant evidence has been published on the significant co-use of BZDs and opiates [54,55]. It should be noted that the prevalence of BZD use among patients maintained on methadone ranges between 51% and 70% and rates close to this range also have been reported for patients on buprenorphine [56] and in active heroin users [24]. In Spain, studies among patients in methadone treatment programs revealed that 46.5% of patients were regularly taking BZDs [57]. The results obtained in our series coincide with the figures cited above, as the simultaneous detection of MOs and BZDs was the most prevalent combination found. Nevertheless, as reported by Reuss et al. [58], it is difficult to discern whether BZDs were a prescribed treatment or drug abuse. In this regard, our results confirm this situation, as the most frequently found pharmacological combination in our series was BZDs plus MOs (mainly tramadol, but also methadone and morphine). Furthermore, our results indicate that most MOs plus BZDs co-users consume mainly intermediate- and long-acting BZDs, and not short-acting BZD, which reinforces previous studies describing that methadone-maintained patients who were regular benzodiazepine users consume long-acting-BZDs (diazepam or flurazepam) to enhance the effects of their daily methadone dose [59]. However, the possibility of serious drug interactions in subjects who concomitantly use these two classes of drugs (opiates and BZDs) has received the attention of researchers and clinicians since the 1970s because of their negative consequences for overall health [24,59]. The high number of deaths with detectable levels of tramadol plus BZDs, suggesting a non-medical use of this opiate, is striking. Concurrent use of tramadol plus BZDs could be of particular concern, as there is a common enzyme for tramadol and benzodiazepine (CYP3A4) that implies a potential for pharmacokinetic interaction. Fatal overdoses of tramadol have been reported especially when taken in combination with BZDs, even at low doses [60]. Furthermore, tramadol, despite being a prescription opiate in Europe, has recently been shown to be present or implicated in at least 300 drug-induced deaths in European countries [61]. The high rates of MO-positive samples and the increasing number of MO plus BZD co-users also seem to indicate an over-exposure to opiates in Spain.

## 5. Conclusions

Overall, our work allows us to conclude that the proportion of psychotropic drug users (excluding ethanol) appears to be high in Spain but remains within the range described for other Western countries. It is possible that more than 40% of the adult population involved in medico-legal issues use/misuse medical psychotropics, and the existence of an evident association between violent deaths and the use of medical psychotropics is particularly worrying. Furthermore, the fact that a quarter of the study population may be BZD users, and that the simultaneous use of several psychotropic drugs (especially the co-use of MOs and BZDs) seems to be evident, is a matter of concern that requires close epidemiological monitoring. Although this pattern of use has also been described in Western populations, these results also suggest that prescription drug abuse could be a worrying reality in Spain and point to the possibility that the use (or misuse) of medical psychotropics could be as relevant, from a medico-legal point of view, as the abuse of illegal psychotropics in developed countries. Our results, although preliminary, reinforce the possibility that toxicological findings from forensic autopsies may be a useful tool for assessing rates of substance use in a population, and indicate the need for future research leading to the identification of at-risk populations and the design of interventions aimed at increasing the control of medical psychotropics use.

## Figures and Tables

**Table 1:** Description of subjects by sociodemographic characteristics and forensic data.

		*n*	(%)
Whole Series		394	100
Date (Year)	2015	124	31.5
	2016	108	27.4
	2017	162	41.1
Gender	Male	298	75.0
	Female	96	24.4
Age (Years)	≤45	156	39.6
	>45	238	60.4
Cause of Death	Traffic accident	42	10.7
	Violent death ^a^	152	38.6
	Suicide	78	19.8
	Others ^b^	122	31.0

^a^ Include firearms, drowning, falls, and overdose; ^b^ include heart diseases, infection, gastrointestinal diseases, sudden death, and unknown causes.

**Table 2:** Mean (and standard deviation) and median (and interquartile range: percentile75–percentile25) in ng/ml, limit of detection (LOD) limit of quantitation (LOQ) in ppb (ng/ml), and frequency of detection of analyzed compounds.

Group	Type	Compound	*n* (%)	Mean (SD)	Median (IR)	LOD	LOQ
Benzodiazepines (BZDs)			95 (24.1)				
	Short-acting BZDs		17 (4.3)				
		Midazolam	17 (4.3)	367.5 (455.7)	180.6 (621.4)	1.50	4.50
	Intermediate-acting BZDs		48 (12.1)				
		Alprazolam	18 (4.5)	226.7 (201.4)	163.7 (254.9)	1.50	4.50
		Bromazepam	0	-	-	6.25	18.75
		Clonazepam	0	-	-	6.25	18.75
		Lorazepam	6 (1.5)	61.5 (32.9)	57.1 (63.8)	6.25	18.75
		Oxazepam	14 (3.5)	295.3 (288.4)	190.8 (314.9)	6.25	18.75
		Temazepam	15 (3.8)	567.5 (663.5)	285.5 (484.4)	1.50	4.50
		Lormetazepam	6 (1.5)	273.2 (396.5)	112.8 (382.4)	6.25	1.75
	Long-acting BZDs		30 (7.6)				
		Nordiazepam	54 (13.7)	1592.3 (3022.2)	610 (1533.8)	1.50	4.50
		Clordiazepoxide	1 (0.3)	43.34 (-)	-	1.50	4.50
		Diazepam	34 (8.6)	288.7 (323.3)	155.9 (304.9)	1.00	3.00
		Flurazepam	1 (0.3)	458.96 (-)	-	1.50	4.50
Z-Drugs	Z-Drugs		8 (2.0)				
		Zolpidem	6 (1.5)	110.7 (203.9)	14.1 (207.5)	1.00	3.00
		Zoplicone	2 (0.5)	180.5 (223.5)	180.5 (-)	6.25	18.75
Antidepressants (ADPs)			41 (10.4)				
	Tri- & Tetra-cyclic ADPs		9 (2.3)				
		Amitriptiline	8 (2.0)	821.01 (796.6)	705.2 (1501.3)	1.00	3.00
		Nortriptiline	9 (2.3)	265.04 (308.3)	193.7 (400.4)	1.00	3.00
		Maprotiline	6 (1.5)	667.8 (789.9)	366.01 (1504.2)	1.00	3.00
	Selective Serotonin Reuptake Inhibitors		33 (8.4)				
		Paroxetine	12 (3.1)	350.9 (514.8)	143.7 (333.3)	6.25	18.75
		Sertraline	8 (2.0)	399.3 (383.2)	249.3 (571.1)	1.00	3.00
		Citalopram	15 (3.8)	232.5 (148.8)	223.8 (184.6)	1.00	3.00
Medical Opiates (MOs)			90 (23)				
		Morphine	33 (8.4)	142.9 (214.9)	71.6 (88.6)	1.00	3.00
		Tramadol	31 (7.9)	1164.2 (3345.4)	178.4 (629.1)	1.00	3.00
		Codeine	7 (1.8)	118.2 (155.7)	23.1 (188.6)	1.00	3.00
		Fentany	8 (2.0)	20.5 (36.8)	5.1 (22.7)	0.50	1.50
		Methadone	28 (7.1)	406.04 (384.4)	309.2 (333.1)	1.00	3.00
Barbiturates			1 (0.3)				
		Fenobarbital	1 (0.3)	352.5 (-)		1.00	3.00

**Table 3:** Relationship between sociodemographic and forensic variables and detected drugs.

Drugs Detected	Gender			Age			Cause of Death				
	Women (%)	Men (%)	*p* Value	≤45 (%)	>45 (%)	*p* Value	Traffic Accident (%)	Violent Death (%)	Suicide (%)	Others (%)	*p* Value
BZDs ^1^ (*n* = 95)	32 (33.7)	63 (66.3)	0.002	38 (40.0)	57 (60.0)	0.064	8 (8.4)	38 (40.0)	25 (26.3)	24 (25.3)	<0.001
Short-acting BZDs (*n* = 17)	4 (23.5)	13 (76.5)	0.049	3 (17.6)	14 (82.4)	0.013	7 (41,2)	9 (52.9)	1 (5.9)	0 (0.0)	0.047
Intermediate-acting BZDs (*n* = 48)	16 (33.3)	32 (66.7)	0.029	26 (54.2)	22 (45.8)	0.665	1 (2.1)	16 (33.3)	15 (31.3)	16 (33.3)	0.004
Long-acting BZDs (*n* = 30)	15 (50.0)	15 (50.0)	1	11 (36.7)	19 (63.3)	0.2	1 (3.3)	13 (43.3)	7 (23.3)	9 (30.0)	0.019
ADPs ^2^ (*n* = 41)	18 (43.9)	23 (56.1)	0.533	13 (31.7)	28 (68.3)	0.028	1 (2.4)	18 (43.9)	15 (36.6)	7 (17.1)	0.001
SSRIs-ADPs ^3^ (*n* = 33)	16 (48.5)	17 (51.5)	1	11 (33.3)	22 (66.7)	0.08	1 (3.0)	17 (51.5)	9 (27.3)	6 (18.2)	0.001
Tri & Tetracyclic ADPs ^4^ (*n* = 9)	3 (33.3)	6 (66.7)	0.508	2 (22.2)	7 (77.8)	0.180	0 (0.0)	1 (11.1)	7 (77.8)	1 (11.1)	0.018
Mos ^5^ (*n* = 90)	31 (34.4)	59 (65.6)	0.004	35 (38.9)	55 (61.1)	0.045	11 (12.2)	37 (41.1)	13 (14.4)	29 (32.2)	<0.001
One drug (*n* = 67)	19 (28.4)	48 (71.6)	0.001	26 (38.8)	41 (61.2)	0.086	9 (13.4)	24 (35.8)	9 (13.4)	25 (37.3)	0.002
Two drugs (*n* = 42)	15 (35.7)	27 (64.3)	0.088	18 (42.9)	24 (57.1)	0.441	5 (11.9)	20 (47.6)	11 (26.2)	6 (14.3)	0.004
≥3 drugs (*n* = 50)	20 (40.0)	30 (60.0)	0.203	18 (36.0)	32 (64.0)	0.065	1 (2.0)	19 (38.0)	17 (34.0)	13 (26.0)	0.001

^1^ BZDs, Benzodiazepines; ^2^ ADPs, Antidepressants; ^3^ SSRIs-ADPs, Selective Serotonin Reuptake Inhibitors Antidepressants (Citalopram, Paroxetine, Sertraline); ^4^ Tri & Tetracyclic Antidepressants (Nortriptiline, Amitriptiline, Maprotiline); ^5^ MOs, Medical Opiates.

**Table 4:** Relationship between number/combination of drugs detected and sociodemographic and forensic variables.

Drugs Detected (Number or Combination of Drugs)	Gender (% Total Samples)			Age (% Total Samples)			Cause of Death (% Total Samples)				
	Women	Men	*p* Value	≤45 Y.	>45 Y.	*p* Value	Traffic Accident	Violent Death	Suicide	Others	*p* Value
BZDs ^1^ + ADPs ^2^ (*n* = 24)	12 (50.0)	12 (50.0)	1	7 (29.2)	17 (70.8)	0.064	0 (0.0)	12 (50.0)	7 (29.2)	5 (20.8)	0.197
BZDs + SSRIs-ADPs ^3^ (*n* = 21)	10 (47.6)	11 (52.4)	1	6 (28.6)	15 (71.4)	0.078	0 (0.0)	11 (52.4)	6 (28.6)	4 (19.0)	0.156
BZDs + Tri & Tetracyclic-ADPs ^4^ (*n* = 3)	2 (66.7)	1 (33.3)	1	1 (33.3)	2 (66.7)	1	0 (0.0)	1 (33.3)	1 (33.3)	1 (33.3)	1
Intermediate-acting BZDs + ADPs (*n* = 12)	6 (50.0)	6 (50.0)	1	6 (50.0)	6 (50.0)	1	0 (0.0)	5 (41.7)	4 (33.3)	3 (25.0)	0.779
Long-acting BZDs + ADPs (*n* = 7)	5 (71.4)	2 (28.6)	0.453	2 (28.6)	5 (71.4)	0.453	0 (0.0)	1 (14.3)	2 (28.6)	4 (57.1)	0.368
BZDs + Mos ^5^ (*n* = 45)	16 (35.6)	29 (64.4)	0.072	18 (40.0)	27 (60.0)	0.233	5 (11.1)	18 (40.0)	10 (22.2)	12 (26.7)	0.052
Intermediate-acting BZDs + MOs (*n* = 24)	8 (33.3)	16 (66.7)	0.152	14 (58.3)	10 (41.7)	0.541	1 (4.2)	9 (37.5)	5 (20.8)	9 (37.5)	0.062
Long-acting BZDs + MOs (*n* = 15)	6 (40.0)	9 (60.0)	0.607	6 (40.0)	9 (60.0)	0.607	1 (6.7)	6 (40.0)	3 (20.0)	5 (33.3)	0.269
MOs + ADPs (*n* = 13)	6 (46.2)	7 (53.8)	1	3 (23.1)	10 (76.9)	0.092	0 (0.0)	9 (69.2)	3 (23.1)	1 (7.7)	0.018
MOs + SSRIs-ADPs (*n* = 11)	5 (45.5)	6 (54.5)	1	3 (27.3)	8 (72.7)	0.227	0 (0.0)	9 (81.8)	2 (18.2)	0 (0.0)	0.035
MOs + Tri & Tetracyclic ADPs (*n* = 2)	1 (50.0)	1 (50.0)	-	0 (0.0)	2 (100.0)	-	0 (0.0)	0 (0.0)	1 (50.0)	1 (50.0)	-

^1^ BZDs, Benzodiazepines; ^2^ ADPs, Antidepressants; ^3^ SSRIs-ADPs, Selective Serotonin Reuptake Inhibitors Antidepressants (Citalopram, Paroxetine, Sertraline); ^4^ Tri & Tetracyclic Antidepressants (Nortriptiline, Amitriptiline, Maprotiline); ^5^ MOs, Medical Opiates.

## Data Availability

Not applicable.
